# Application of Absorption and Scattering Properties Obtained through Image Pre-Classification Method Using a Laser Backscattering Imaging System to Detect Kiwifruit Chilling Injury

**DOI:** 10.3390/foods10071446

**Published:** 2021-06-22

**Authors:** Zhuo Yang, Mo Li, Andrew R. East, Manuela Zude-Sasse

**Affiliations:** 1 MAF Digital Lab, Massey University, 4472 Palmerston North, New Zealand; Z.Yang1@massey.ac.nz (Z.Y.); A.R.East@massey.ac.nz (A.R.E.); 2 School of Food and Advanced Technology, Massey University, 4410 Palmerston North, New Zealand; 3 Leibniz Institute for Agricultural Engineering and Bioeconomy (ATB), 14469 Potsdam, Germany

**Keywords:** non-destructive detection, diffusion theory, spatially-resolved, optical phantom

## Abstract

Kiwifruit chilling injury (CI) damage occurs after long-term exposure to low temperature. A non-destructive approach to detect CI injury was tested in the present study, using a laser backscattering image (LBI) technique calibrated with 56 liquid phantoms for providing absorption coefficient (µ_a_) and reduced scattering coefficient (µ_s_’). Calibration of LBI resulted in a true-positive (TP) classification of 91.5% and 65.6% of predicted µ_s_’ and µ_a_, respectively. The optical properties of ‘SunGold™’and ‘Hayward’ kiwifruit were analysed at 520 nm with a two-step protocol capturing pre-classification according to the LBI parameters used in the calibration and estimation with the Farrell equation. Severely injured kiwifruit showed white corky tissue and water soaking, reduced soluble solids content and firmness measured destructively. Non-destructive classification results for ‘SunGold™’ showed a high percentage of TP for severe CI of 92% and 75% using LBI parameters directly and predicted µ_a_ and µ_s_’ after pre-classification, respectively. The classification accuracy for severe CI ‘Hayward’ kiwifruit with LBI parameter was low (58%) and with µ_a_ and µ_s_’ decreased further (35%), which was assumed to be due to interference caused by the long trichomes on the fruit surface.

## 1. Introduction

Kiwifruit chilling injury (CI) is a physiological response that causes cell damage during long term storage at low temperature [1]. Symptoms of kiwifruit CI are complex and can be observed on the exocarp and mesocarp tissue. Initial symptoms are only visible when cutting the fruit. Early-stage symptoms of CI can be observed as white spots in the mesocarp tissue at the stylar end, which develop to severe symptoms of granulation and water soaking [2]. The incidence and severity of kiwifruit CI are affected by harvest maturity, particularly early harvest fruit are more susceptible to CI [3]. Rapid cooling [4], lower temperature during long-term storage [5] and exposure to ethylene [6] can also exacerbate CI incidence. CI can affect fruit quality, external appearance, respiration rate, and ethylene production [7]. Failure to detect kiwifruit CI may, therefore, result in a significant fruit loss during storage and marketing [8].

At present, kiwifruit CI is assessed visually by cutting a subsample of fruit from a grower line batch before exporting, assuming that the sample is representative of the entire population. A reliable non-destructive technique is desired for kiwifruit CI detection enabling inline grading of entire batches of fruit before marketing. Optical measuring principles have been demonstrated to non-destructively detect fruit internal disorders previously. Magnetic resonance imaging (MRI) techniques were applied to rapidly detection moth infestation [9], bruising [10], and mealiness [11] of apple. Soft X-ray imaging was applied to classify bruise [12] and watercore [13]. Thermal imaging has been used to detect apple bruising [14]. However, MRI has been confirmed for analysing the structure of fruit tissue including water soaking, but is still very expensive and the analysis is slow, which makes the technique less suitable for sorting fruit. Soft X-ray imaging requires advanced segmentation to distinguish new bruises within apple tissue, which might be challenging for granulation detection for intact kiwifruit. However, this needs to be further studied. Although IR has advantages such as a large measurement area and suitability for moving fruit on the sorting line, IR typically requires temperature adjustment while recording the thermal activities in the sample of interest and the thermal camera can be costly. This measuring scenario may create challenges in industrial applications. Additionally, IR seems to be more suitable at detecting more severe tissue damage caused by mechanical damage (e.g., bruised tissue) which would alter thermal emissivity of the fruit. For chilling injury, especially when symptoms are mild to moderate (i.e., granulation of tissue without water soaking appearance) the application may not be feasible.

Near infrared (NIR) spectroscopy has been applied earlier to detect CI in fruit and vegetables [15]. Wang et al. segregated sound kiwifruit and kiwifruit with CI using NIR spectral information and reported a stronger separation at the stylar end compared to the analysis in the equatorial region [16]. Kemsley et al. investigated NIR diffuse optical tomography at 689 nm and reported internal defect detection in potatoes [17]. Hyperspectral image analysis has been widely investigated for evaluating horticultural products [18]. Since CI involves pigment changes for some crops, the capacity of spectral data in CI detection appears reasonable. In Wang et al.’s study [16], kiwifruit were segregated in severe CI cases when discolouration had already occurred. Cen et al. applied selected wavelengths and image features to detect CI symptoms of brown spots in cucumber and achieved 100% classification accuracy for a two-class classification [19]. Hyperspectral imaging at selected wavelengths was used for CI detection in apples, obtaining 98.4% overall classification accuracy [20]. So far, no early detection of CI in kiwifruit has been reported.

Limitations of ‘point’ NIR spectroscopy and hyperspectral imaging technique include the high cost for hardware and complex data analysis. Wavelength selection and binning techniques have been explored to reduce the data processing time and cost of the sensor hardware since the selection of relevant wavelengths enables a multi-spectral sensor setup with less expensive hardware. However, a drawback for obtaining fruit information from spectroscopy is related to the varying cell wall structure of the sample, which affects the spectral intensities measured due to its effect on the scattering events in the tissue. The apparent spectral-optical information represents the sum signal of absorption and scattering. Li et al. investigated the postharvest quality of kiwifruit using the NIR technique to predict kiwifruit storability and reported poor prediction performance of kiwifruit firmness [21]. ElMasry et al. predicted Red Delicious apple firmness using hyperspectral imaging and obtained high root mean square error (8.26 N and 9.40 N for training and validation, respectively) despite a 90% classification accuracy [20]. Walsh et al. explained in their review that errors could occur when using spectral reflectance data to predict fruit quality, because the sum signal changes with both chemical composition and physical properties [15]. CI at the early stage represents the appearance of granular tissue that may mainly affect the scattering properties of the fruit. Therefore, a non-destructive technique with the potential for measuring mainly the scattering property of the fruit tissue is desired.

A laser backscattering image (LBI) system is a multi-spectral system based on preselected wavelengths. The sum signal of absorption and scattering is recorded via an image of diffusely reflected light from the laser incident point till attenuation of light in the tissue. This method has been identified as providing information that distinguishes absorption and reduced scattering properties using the radial light attenuation profile followed by appropriate data analysis [22]. The decoupled absorption coefficient (µ_a_) and reduced scattering coefficient (µ_s_’), which are related to chemical composition and cell wall structure, may be correlated with internal disorder symptoms of water soaking, discolouration and corky tissue. In previous research, fruit’s optical properties were predicted via a curve fitting process in the diffusion model developed by Farrell et al. [22]. In this case, LBI may be more useful as it is potentially more sensitive to detecting changes in the scattering of photons as a result of tissue granulation. Additionally, the cost of LBI system components (laser diode and CCD camera) is reasonable compared to other non-destructive systems. LBI system is easy to be implemented on existing fruit sorting/grading line.

LBI profiles were applied to identify CI in banana and reported LBI parameters were correlated with fruit quality parameters that could potentially indicate pigment changes due to CI [23]. Lu and Peng used a multi-spectral scattering profile with Lorentzian distribution to predict peach firmness [24]. A broader scattering profile of soft fruit than firm fruit was observed, and Lorentzian parameters were linearly related to fruit firmness. Van Beers et al. applied relative reflection profile data to predict apple maturity and observed good prediction performance of at-harvest maturity and starch level, but poor performance for SSC and firmness prediction [25]. Peng and Lu approached an improvement of firmness analysis by adding shape correction [26]. However, the decoupling of µ_a_ and µ_s_’ remains a major challenge for LBI when applied to predict fruit quality. Baranyai and Zude investigated the apple scattering coefficient from LBI image via Monte Carlo simulation and reported that LBI profiles changed with induced bruising symptoms [27]. However, it was also reported that simulation of high scattering—low absorbing and low scattering—high absorbing samples were hardly distinguishable. Zude-Sasse et al. validated optical properties of pear using the destructive photon density wave (PDW) method and found that measuring uncertainty could be introduced during the curve fitting process without limiting the range of either µ_a_ or µ_s_’ according to known values of one variable by means of destructive analysis [28]. Therefore, a calibration of the LBI system and the according pre-classification of µ_a_ or µ_s_’ ranges before the fitting process could potentially reduce the measuring uncertainty.

This so-called metamodeling was introduced for bridging the gap between the mathematical model and real-world fruit by using the LBI parameter of liquid phantoms to provide a reference matrix for pre-classifying the ranges of optical properties of fruit samples [18,28]. Liquid phantoms are turbid media with absorbers at a known concentration and lipids serving as scatterers. The optical properties were estimated and validated by Aernouts et al. and Watté et al. [29,30]. Yang et al. investigated kiwifruit optical properties using solid phantoms and found that the range of µ_a_ (cm^−1^) ∈ [0, 1.2] and µ_s_’ (cm^−1^) ∈ [0, 15] should be resolved at a higher resolution for kiwifruit compared to currently reported metamodels [31].

The objectives of the present study were to (1) develop a pre-classification model for segregating LBI images with µ_a_ or µ_s_’ using liquid phantom profiles with enhanced resolution of µ_s_’ and µ_a_; and (2) investigate whether optical properties calculated after pre-classification could be used to distinguish sound kiwifruit and kiwifruit with CI symptoms.

## 2. Materials and Methods

### 2.1. Fruit Materials

‘Hayward’ kiwifruit (*Actinidia chinensis var. deliciosa*, *n* = 400) were harvested at 8 maturity stages from 6 April–1 June 2020 at weekly intervals. Kiwifruit were harvested from one commercial orchard and stored at −0.5 °C for 22 weeks to induce CI symptoms. LBI data and CI symptoms were measured after storage.

Cool stored ‘SunGold™’ kiwifruit (*A. chinensis var. chinensis*, *n* = 396) were delivered to Massey University on August 14th 2020 from 3 growers after 20 weeks of storage. ‘SunGold™’ kiwifruit samples were stored at 1 °C cold room for 30 days and were measured subsequently after warming up to 20 °C overnight.

### 2.2. LBI Image Capture

**Figure 1:**
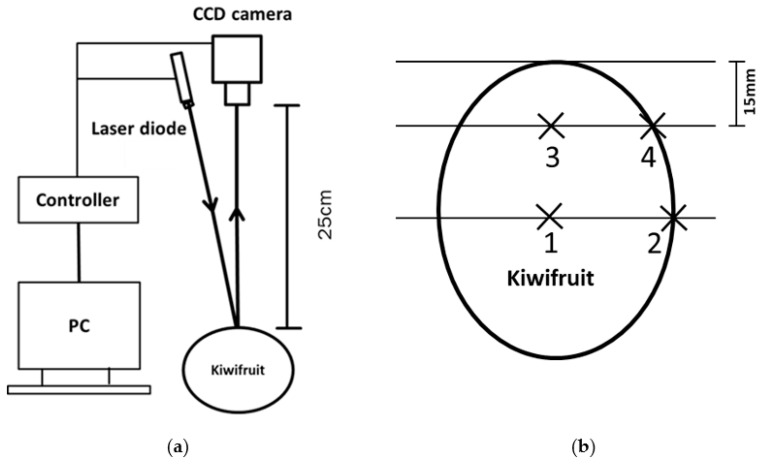
(**a**) Laser backscattering imaging system; (**b**) kiwifruit assessment. Cross-marks of the laser pointer focus (3, 4 in the stylar end region; 1, 2 in the equatorial region), cut line to assess kiwifruit chilling injury.

The LBI imaging system (Figure 1a) was assembled at Massey University, New Zealand. The system included the light source with a laser diode (FP-D-DIG-520-17-C-F250-USB, Laser Components, Germany) emitting at 520 nm with 6.8 mW of power output, a charge-coupled device (CCD) camera (MER-131-210U3M NIR, China Daheng Group, China) providing resolution of 1280 × 1024 pixels, equipped with F1.4 aperture zoom lens and 10–40 mm focal length (12VG1040 ASIR-SQ, Tamron Co. Ltd., Saitama, Japan), and a desktop computer to control the light source and camera. An electrical moving table was used to adjust the sample position to maintain a distance of 25 cm between the highest point of the fruit surface and the camera.

Non-destructive LBI measurement was conducted at 520 nm, which is the absorption peak of carotenoids. Each kiwifruit was placed longitudinally on the moving table. Kiwifruit LBIs were taken at 4 positions with 2 each (at 90° apart) on the equatorial and the stylar end region (1.5 cm from the end) (Figure 1b). The 4 positions corresponded to the focus points of the laser pointer when imaging the samples. A custom written Labview software (National Instruments, Austin, TX, USA) was used to control the system. Each resulting LBI was the average image of 10 images acquired as 0.5 s per image. Acquisition of the images was done in a dark room and the measuring parts were secured inside a black cage to prevent interfering stray light and avoid exposure to laser light.

### 2.3. Kiwifruit Quality Assessment

Kiwifruit fresh weight was measured using an electronic balance (TW423L, Shimadzu, Japan). Kiwifruit flesh firmness (FF) was determined from penetration tests using a penetrometer (Willowbank Electronics Ltd., Napier, New Zealand) with a standard 7.9 mm diameter convex Effegi probe at a speed of 8 mm s^−1^ to 8 mm depth. Before the measurement, 2 mm of skin slice was removed at two equatorial positions (90° apart). Kiwifruit FF was recorded as the average of the two readings. The SSC (%) of kiwifruit was measured by a refractometer (PR-32α, Atago, Tokyo, Japan) at 20 °C. Kiwifruit samples were cut into half at the equator and juice extracted by hand from the stylar end half. Kiwifruit longitudinal profile (lp) was calculated using kiwifruit length, minor diameter and major diameter [32].

### 2.4. Kiwifruit CI Assessment

Kiwifruit samples were cut along the marked position at the stylar end (Figure 1b) for manual observation of the CI severity. The CI assessment was carried out at the four locations matching the LBI measurement area. The severity score was obtained using a scale provided by Wang et al. and was recorded as CI_se_ and CI_eq_ for the stylar end and equatorial section of kiwifruit, respectively [16].

### 2.5. LBI Profile Analysis

**Figure 2:**
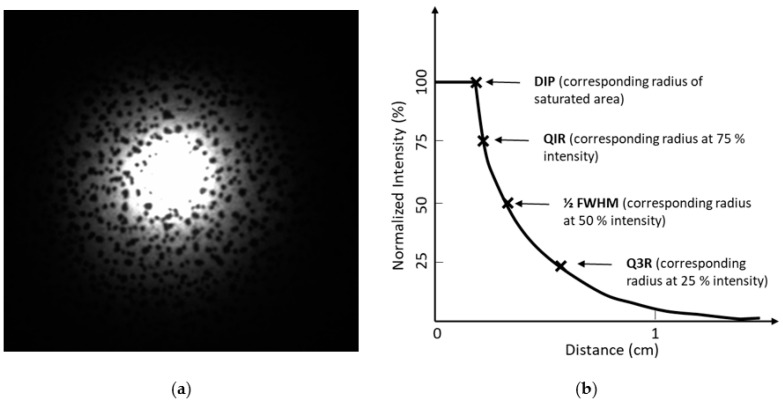
(**a**) ‘SunGold™’ kiwifruit LBI; (**b**) transformed diffusion profile from laser backscattering image (LBI). LBI parameters were the radius of the saturated area (distance to incident point, DIP), the radius at 75% of maximum intensity (Q1R), double the radius at 50% of maximum intensity (full width half maximum, FWHM), the radius at 25% of maximum intensity (Q3R), and the negative slope (SLP) of the linear regression model built with log-transformed profile data between Q1R and Q3R.

Raw LBI (Figure 2a) was analysed by R (version 3.6.0, R Foundation for Statistical Computing, Vienna, Austria) running in RStudio (version 1.0.153, RStudio Inc., Boston, MA, USA). An average of 10 LBI were used in further analysis. In the geometric calibration, one pixel represented 0.01015625 · 0.01015625 cm^2^. The raw image was transformed into a grey-scale matrix using the ‘readbitmap’ package (version 0.1.5). The backscattering intensity matrix was computed into LBI light attenuation profiles. LBI parameters were extracted as the radius of the saturated area (distance to incident point, DIP), the radius at 75% of maximum intensity (Q1R), double the radius at 50% of maximum intensity (full width half maximum, FWHM), the radius at 25% of maximum intensity (Q3R), and the negative slope (SLP) of the linear regression model [31] built with log-transformed profile data between Q1R and Q3R (Figure 2b).

LBI parameters were extracted from images obtained at the stylar end (se)—positions 3 and 4, Figure 1, and equatorial region (eq)—positions 1 and 2, Figure 1, respectively, as well as from all images of the whole fruit (wf)—mean of positions 1–4, Figure 1**.** In a three-class classification, LBI and kiwifruit were segregated into Sound (free from CI symptoms), Moderate (CI symptoms of granulation but without water soaking) and Severe (CI symptoms of both granulation and water soaking).

### 2.6. Liquid Phantoms and Pre-Classification Model

A total of 56 liquid phantoms were prepared for pre-classification model calibration. Liquid phantoms were made by Intralipid^®^ 20% (batch 80NC095, Fresenius Kabi, Germany) and Naphthol Blue Black (NBB) (Sigma-Aldrich, St. Louis, MO, USA) 5 mM stock solution. The absorbers of liquid phantoms are ink particles, and the scatterers are lipid particles. The designed phantom set had 4 levels of scattering at 3.12%, 5.2%, 7.28% and 9.36% of Intralipid^®^, and 14 levels of absorption between 0–0.65% of NBB at 0.05% interval. Phantom solutions were made using 250 mL volume flasks then transferred into 280 mL black plastic containers (41 × 118 mm) for LBI acquisition. Liquid phantom LBI images were obtained immediately after preparation of phantoms by placing the container in the centre of the moving table of LBI system. Liquid phantom LBI images were pre-processed to remove the reflection spot within the diffusion area. LBI diffusion profiles were extracted from the pre-processed picture.

The absorption coefficient of the liquid phantom µ_a_* was measured using a UV-visible recording spectrophotometer (UV–16 A, SHIMADZU, Japan) at different NBB concentrations. Measured µ_a_* was linearly correlated (R^2^ = 0.998) with NBB concentration maybe due to minor handling errors.

µ_a_* = −0.0816 A − 0.0624 (1)

in which, µ_a_* of the phantom referred to the calculated absorption coefficient of NBB solution according to measured absorbance, A (Equation (1)). This equation was built according to measured data.

A minimum µ_a_* was set as 1.e^−10^ as µ_a_* ≠ 0 was a condition in the curve fitting process [22]. The estimated µ_a_ range (Equation (2)) was then calculated as

µ_an_* ∈ [µ_an_ − (µ_an_ − µ_an-0_._05_)/2, µ_an_ + (µ_an+0_._05_ − µ_an_)/2] (2)

in which, µ_an_* is µ_a_* with *n*% of NBB.

The estimated reduced scattering coefficient of the liquid phantom µ_s_‘* was measured according to Watté et al. at different Intralipid^®^ 20% concentration [30]. A linear regression (R^2^ = 0.99) was found using Intralipid^®^ 20% concentration and estimated µ_s_’* described by

µ_s_’* = − 0.003272 + 2.985451 · C · 20% (3)

in which, µ_s_’* represented the estimated reduced scattering coefficient of liquid phantoms and C was Intralipid^®^ concentration. The resulting µ_s_’* levels are given in Table 1, providing µ_s_’ ranges calculated with Equation (2).

Liquid phantom extracted LBI parameters, µ_a_* and µ_s_’* (Equation (3)) were used to calibrate the LBI system and provide the data base for the pre-classification model using flexible discriminant analysis (FDA) with leave-one-out cross-validation to predict the class of µ_a_ and µ_s_’. Kiwifruit LBI parameters were extracted, and attenuation profiles were pre-classified according to the reference classes set by the phantoms. Then, the predicted µ_a_ (cm^−1^) and µ_s_’ (cm^−1^) were subsequently calculated with Farrell’s equation considering the ranges from pre-classification. In which µ_a_*, µ_s_’* were the known values and µ_a_ range (data not shown), µ_s_’ ranges (Table 1) were the prediction boundaries for each pre-predicted class in the curve fitting process.

### 2.7. Data Analysis

The quantitative comparison of LBI parameters (DIP, Q1R, FWHM, Q3R, SLP) and kiwifruit optical parameters (µ_a_, µ_s_’) for sound, moderated and severe samples were subjected to analysis of variance (ANOVA). Tukey’s HSD test was applied to test the significance of the variable effect. As the goal is to classify sound and CI kiwifruit, their optical properties were analysed via FDA in ‘mda’ package (version 0.4–10). A confusion matrix was presented to evaluate the performance of the prediction model.

Data were analysed directly and with 10 subsample sets with the same number of severe CI kiwifruit from all categories were chosen by selecting random seeds used to deal with the unbalanced population of sound, moderated and severe CI samples. A confusion matrix was calculated in R and true positives (TP) given. TP are kiwifruit correctly predicted for each severity.

Two-class segregation was also investigated. Fruit from sound and moderate groups were pooled together and a model was developed to segregate them from the severe group. Because of the large number of fruit in the sound group, data balancing was carried out by assigning the same number of severe to sound and moderate kiwifruit through sub-sampling, whilst keeping the same ratio of sound and moderate fruit (‘SunGold™’ = 5:1, ‘Hayward’ = 4:5) in the balanced population. F_1_ score was selected, as a reliable indicator for unbalanced data sets, to predict kiwifruit with severe CI, when comparing the results from the entire population in the two-class segregation. F_1_ was calculated with performance metrics: precision (P) and recall (R) (Equations (4)–(6)).

(4) $$P\;=\frac{\mathrm{TP}}{\mathrm{TP}+\mathrm{FP}}$$

(5) $$R\;=\frac{\mathrm{TP}}{\mathrm{TP}+\mathrm{FN}}$$

(6) $$F_{1}=2\cdot \frac{P\cdot R}{P+R}$$

in which, TP were kiwifruit correctly predicted with severe CI, FP were kiwifruit mistakenly predicted with severe CI, TN were kiwifruit correctly predicted as sound or moderate CI and FN were kiwifruit mistakenly predicted as sound or moderate CI.

## 3. Results and Discussion

### 3.1. Pre-Classification Model

**Figure 3:**
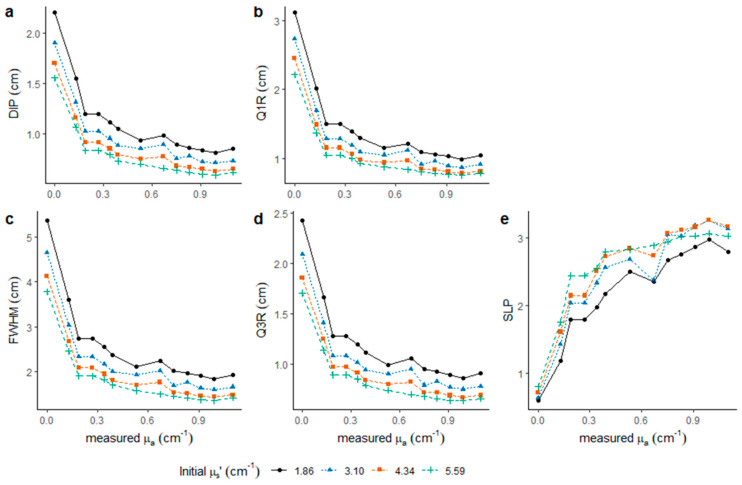
Parameters extracted from LBI profiles at 520 nm after imaging processing considering 4 known µ_s_’*. LBI parameters were the (**a**) radius of the saturated area (DIP), (**b**) the radius at 75% of maximum intensity (Q1R), (**c**) double the radius at 50% of maximum intensity (FWHM), (**d**) radius at 25% of maximum intensity (Q3R), and (**e**) slope of the linear regression model (SLP) built with log-transformed profile data between Q1R and Q3R.

LBI diffusion profiles of liquid phantoms were extracted considering varying levels of absorption and scattering. Parameters of LBI profile were extracted (Figure 3), showing that values of DIP, Q1R, FWHM and Q3R decreased at high measured µ_a_*, but also at high scattering level. This same result was previously reported for banana, showing that FWHM measured at 660 nm appeared lower at early green ripening stages with enhanced chlorophyll content compared to yellow ripe fruit with decreased chlorophyll content. The latter resulting in reduced µ_a_ at 660 nm [23]. The difference in LBI parameters among different scattering levels was somewhat stable at all measured µ_a_* levels as illustrated by the clear separation in most cases, while the parameters decreased quickly when µ_a_* changed from 0 to 0.19 cm^−1^.

**Figure 4:**
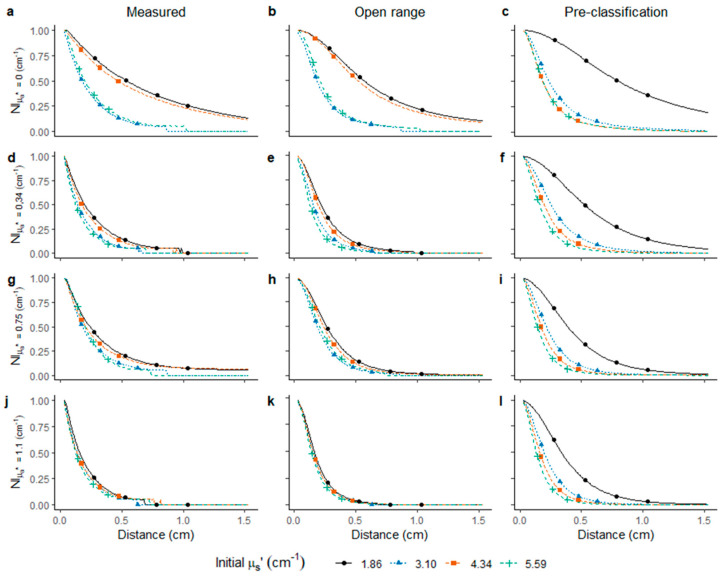
Diffusion profile of liquid phantoms obtained by LBI measurement at 520 nm (**a**,**d**,**g**,**j**); simulated profiles using Farrell equation with measured µ_a_* and predicted µ_s_’ of phantoms without pre-classification (**b**,**e**,**h**,**k**); simulation using Farrell equation with measured µ_a_* and predicted µ_s_’ of phantoms after pre-classification (**c**,**f**,**i**,**l**). Dots mark intensity at 75%, 50% and 25%.

In the present study, measured LBI profiles of phantoms with different µ_s_’* values appeared separated, suggesting that classification according to the range (Equation (3)) was possible (Figure 4a,d,g,j). However, the difference among phantoms with varying µ_s_’* became less obvious with increasing absorption levels. In an earlier study, the LBI profiles and its µ_a_ and µ_s_’ of ‘Conference’ pear showed the same trend with increasing µ_a_ and decreasing µ_s_’ [33]. Simulation of the profiles was done according to Farrell equation with open ranges, without setting the ranges according to the calibration, but with known µ_a_*. Surprisingly, the knowledge on µ_a_* resulted in no clear separation according to the optical properties (Figure 4b,e,h,k). However, when simulating the profiles with known µ_a_* values and pre-classification ranges of µ_s_’ according to the calibration with liquid phantoms resulted in clear separation of the simulated profiles (Figure 4c,f,i,l).

**Figure 5:**
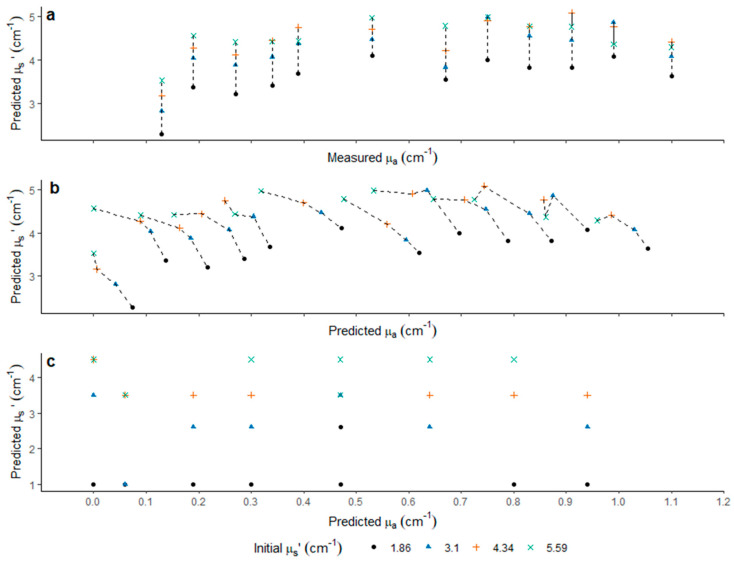
Optical properties prediction of phantom at 4 known µ_s_’* by means of (**a**) destructively measured µ_a_* and predicted µ_s_’ directly (µ_s_’ = open range) according to Farrell equation; (**b**) predicted µ_a_ and µ_s_’ (both open range); (**c**) predicted µ_a_ and µ_s_’ (both after pre-classification).

When simulating the diffusion profiles based on µ_a_* and predicted µ_s_’*, obtained by fitting of the measured LBI profile directly with the Farrell equation with one known variable, prediction of µ_s_’ was possible for µ_a_* < 0.7 cm^−1^. However, an under-estimation of µ_s_’ occurred. With enhanced µ_a_*, the separation of µ_s_’ showed an increased uncertainty (Figure 5a). Predicted µ_a_ calculated from predicted µ_s_’ (Figure 5b) showed an under-estimation of µ_a_ with increasing µ_s_’, resulting in diagonal parallel lines shifting towards the top left for the different µ_s_’ levels at the same µ_a_* level (Figure 5b). A similar observation of underrepresentation of µ_a_ and over-representation of µ_s_’ was reported by Yang et al. using solid phantoms to build the pre-classification model with µ_a_* and µ_s_’* ranging between 0.1–1.3 and 2.0–22.8 cm^−1^, respectively [31]. When using the FDA pre-classification based on LBI parameters, µ_a_ and µ_s_’ were separated non-destructively. Although prediction bias was found, a potential to use LBI parameters to build a pre-classification model aimed at predicting optical properties (Figure 5c) was confirmed in the present study. However, the prediction error observed in Figure 5c is high. Previous metamodel approaches used hyperspectral imaging system [29] and liquid phantoms with µ_a_ at 550–700 nm and 900–950 nm. A large variation was observed, when µ_a_ ranged between [0, 0.5] cm^−1^, while µ_s_’ prediction reached R^2^ = 0.997 and RMSE = 0.226 cm^−1^. Such findings were achieved when the µ_s_’ levels captured a high range between [3.8, 20] cm^−1^ [29]. A clear level of separation of µ_a_ and µ_s_’ was observed for large ranges of liquid phantom sets, in which µ_a_ and µ_s_’ were between 0–14 and 0–275 cm^−1^ [34].

At lower absorption levels (µ_a_* ≤ 0.59 cm^−1^), segregation among scattering levels could be observed regardless of the shifting of µ_s_’ with increasing µ_a_*. However, the classification of µ_s_’ is less accurate when µ_a_* > 0.59 cm^−1^, which might be expected due to the similarity of LBI profiles at higher µ_a_* (Figure 4). Higher accuracy was obtained for predicted µ_a_ at lower µ_s_’. The predicted optical properties (Figure 5c) were not separated well. However, pre-classification showed improved prediction for µ_s_’, close to the known value µ_s_’*, with pre-classification (Table 1). Similar results were reported when applying time resolved spectroscopy for low µ_s_’ and high µ_a_. When µ_s_’ > 20 cm^−1^ and µ_a_ < 0.2 cm^−1^, the model performance was high, and error still less than 20% when µ_s_’ > 5 cm^−1^ [35].

As described previously [22], curve fitting with Farrell equation is reliable when µ_a_ << µ_s_’, while in the current liquid phantom set, µ_s_’ was studied within a more narrow range between [1.86, 5.59] cm^−1^ with enhanced resolution compared to earlier research works [34,35]. Consequently, limited prediction capacity may have been expected in the range, which is, however, relevant for kiwifruit. The range of optical properties of kiwifruit has been described as µ_a_ between 0–0.4 cm^−1^ and µ_s_’ < 8 cm^−1^ within visible-NIR region [36]. Thus, the set of phantoms used in the present study covered the relevant range. The prediction of µ_s_’ at µ_a_* = 0 or prediction of µ_s_’ with an open range without pre-classification, resulted in high difference from actual µ_s_’* (Table 1). However, when using the pre-classification, µ_s_’ was predicted in the correct range.

The pre-classification model was cross-validated with the same set of liquid phantom LBI profiles and the associated µ_a_* and µ_s_’*. The overall accuracy after pre-classification was 65.6% and 91.5% considering µ_a_ and µ_s_’, respectively (Table 2). Classification accuracy of µ_s_’ (Table 2) was higher than for µ_a_, which may be due to the small absorption class intervals used in the classification model as indicated by LBI parameters in Figure 2. The classification accuracy of predicted µ_s_’ without pre-classification was worse than that using LBI parameters because without limiting the range, predicted µ_s_’ tends to be over-estimated at lower µ_s_’ levels (Table 1). This indicated that µ_s_’ range is required to be known when using Farrell’s equation to get more accurate estimates of µ_s_’ values.

### 3.2. Kiwifruit Segregation

#### 3.2.1. Kiwifruit LBI Profiles

CI had an effect on flesh firmness and SSC for kiwifruit in severity categories (Table 3). For both cultivars, sound kiwifruit had higher flesh firmness and SSC compared to damaged fruit. This may be explained by advanced ripening due to enhanced ethylene production [6] caused by CI or the disintegration of cell structures and associated tissue damage as a result of CI [37]. Since the number of samples appeared unbalanced, in further analysis subsamples of the entire data sets were used.

**Figure 6:**
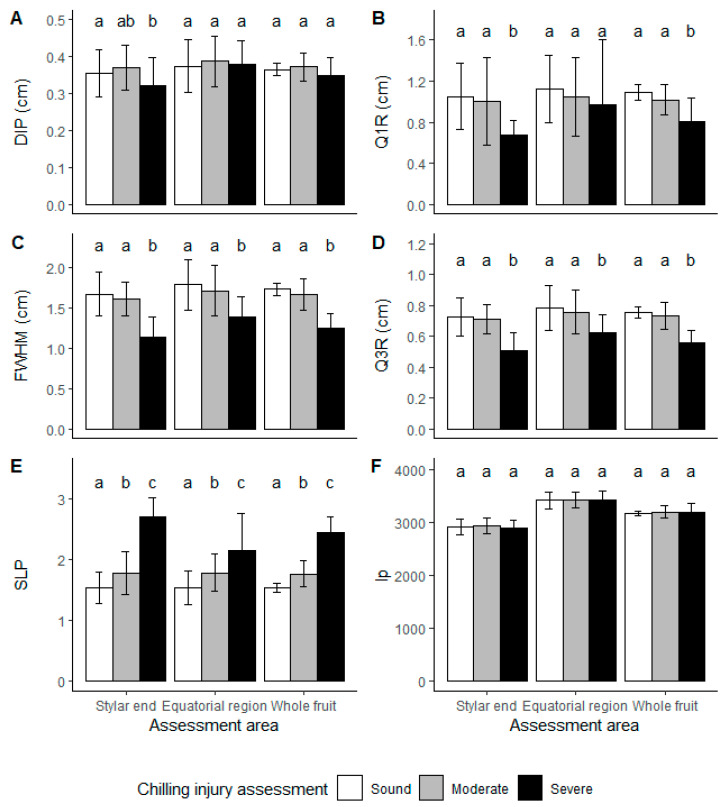
‘SunGold™’ kiwifruit LBI profile parameters for segregation of chilling injuries (CI) in stored kiwifruit. White, grey and black bars represent CI assessed as sound (free from CI symptoms), moderate (CI symptoms of granulation but no water soaking) and severe (both CI symptoms of granulation and water soaking) kiwifruit. Lower case letters represent a significant difference between three CI severities by Tukey HSD (*p*-value < 0.05) using LBI taken from two locations or whole fruit. Error bars represent the standard deviation. LBI parameters were (**A**). the radius of the saturated area (distance to incident point, DIP), (**B**). the radius at 75% of maximum intensity (Q1R), (**C**). double the radius at 50% of maximum intensity (full width half maximum, FWHM), (**D**). the radius at 25% of maximum intensity (Q3R), (**E**). and the negative slope (SLP) of the linear regression model built with log-transformed profile data between Q1R and Q3R. (**F**). kiwifruit longitudinal profiles (lp) were compared at positions where LBI taken.

When comparing LBI parameters, Q1R, FWHM and Q3R were reduced in kiwifruit showing severe CI (Figure 6). This was found regardless of the acquisition position of the LBI on the fruit. The SLP was different among all 3 CI severity classes, suggesting that LBI profiles could be employed to segregate kiwifruit according to the CI severity. Such assumption is supported by the high differences of LBI profiles and corresponding LBI parameters due to changes in optical properties (Figure 4). DIP remained constant for all samples, possibly because DIP describes the saturated area of LBI image, which is mainly affected by the laser power output. Despite the fact that CI symptoms developed from the stylar end, the position of LBI measurement hardly showed any effect on the differences of LBI parameters.

**Figure 7:**
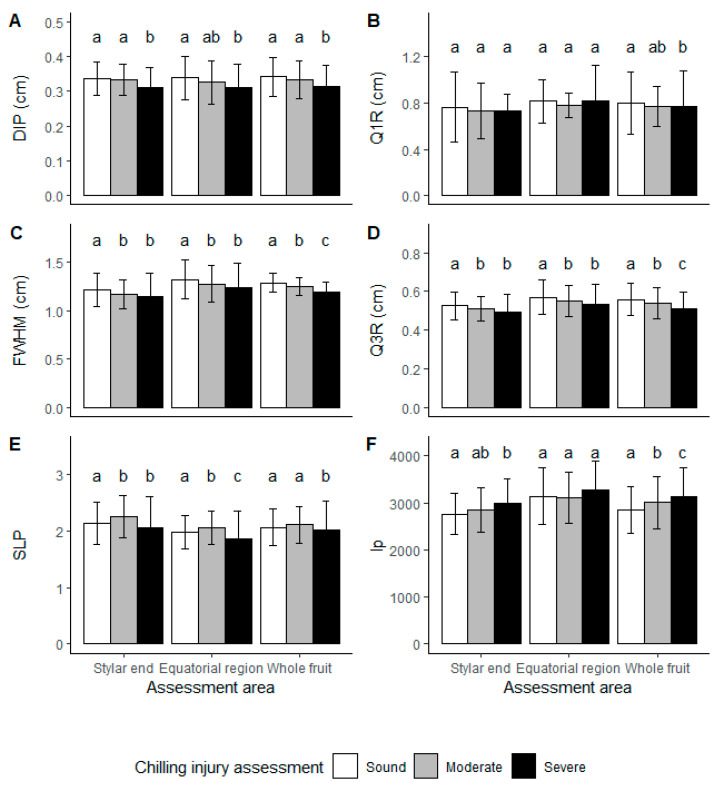
‘Hayward’ kiwifruit LBI profile parameters for segregation of chilling injuries (CI) in stored kiwifruit. White, grey and black bars represent CI assessed as sound (free from CI symptoms), moderate (CI symptoms of granulation but no water soaking) and severe (both CI symptoms of granulation and water soaking) kiwifruit. Lower case letters represent a significant difference between three CI severities by Tukey HSD (*p*-value < 0.05) using LBI taken from two locations or whole fruit. Error bars represent the standard deviation. LBI parameters were (**A**). the radius of the saturated area (distance to incident point, DIP), (**B**). the radius at 75% of maximum intensity (Q1R), (**C**). double the radius at 50% of maximum intensity (full width half maximum, FWHM), (**D**). the radius at 25% of maximum intensity (Q3R), (**E**). and the negative slope (SLP) of the linear regression model built with log-transformed profile data between Q1R and Q3R. (**F**). kiwifruit longitudinal profiles (lp) were compared at positions where LBI taken.

For ‘Hayward’ kiwifruit, similar results were observed (Figure 7). For LBI images, differences were found in FWHM, Q3R and negative SLP, which may be applied to segregate kiwifruit based on CI categories. Unlike ‘SunGold™’, DIP was different for severe CI kiwifruit for ‘Hayward’ (Figure 7A). Additionally, a longitudinal profile describing the shape difference was observed for different CI categories at the stylar end (Figure 7F). Shape differences may occur, because the kiwifruit were ungraded and possible correlation between size and CI susceptibility may be a subject to future studies. In general, the least advanced kiwifruit are the smaller size fruit in a batch, which are more susceptible to CI [3].

The LBI parameters DIP and SLP differed for severe CI kiwifruit in comparison to the categories of sound and moderate CI fruit. Furthermore, FWHM, Q3R and longitudinal profile were different for all three severities, suggesting a potential to use LBI parameters to segregate ‘Hayward’ kiwifruit with different CI severity. The changes in LBI parameters could result from quality difference (FF and SSC) between CI classes (Table 3). However, granulation and water soaking should be the more dominant effects.

#### 3.2.2. Kiwifruit LBI Optical Properties

**Figure 8:**
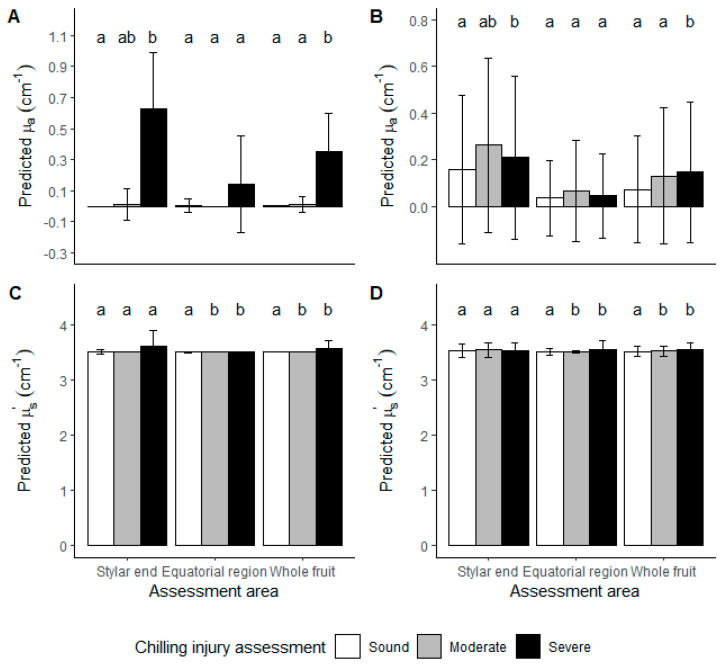
‘SunGold™’ kiwifruit LBI µ_a_ (**A**) and µ_s_’(**C**) and ‘Hayward’ kiwifruit LBI µ_a_ (**B**) and µ_s_’ (**D**) for segregation of chilling injuries in stored kiwifruit. White, grey and black bars represent CI assessed as sound (free from CI symptoms), moderate (CI symptoms of granulation but no water soaking) and severe (both CI symptoms of granulation and water soaking) kiwifruit. Lower case letters represent a significant difference between three CI severities by Tukey HSD (*p*-value = 0.05) using LBI taken from two locations or whole fruit.

Optical properties of kiwifruit were predicted employing LBI parameters for pre-classification before using Farrell equation for fitting the µ_a_ and µ_s_’ to the non-destructively measured LBI diffusion profile. A large standard deviation was observed for predicted µ_a_ for both cultivars. Predicted µ_a_ was different for sound kiwifruit for ‘SunGold™’ when measuring at stylar end and equatorial region (Figure 8A), whereas no difference was observed for ‘Hayward’ (Figure 8B). CI symptoms include pigment changes, such as discolouration of the skin in the outer pericarp region near the stylar end, could be related to µ_a_, thus higher µ_a_ was observed for kiwifruit with CI. However, for ‘Hayward’ kiwifruit, CI symptoms did not involve pigment changes on the skin and, consistently, no difference in predicted µ_a_ was observed in the ‘Hayward’ data set. Enhanced predicted µ_s_’ was observed for ‘SunGold™’ kiwifruit with CI at the stylar end region and ‘Hayward’ kiwifruit with CI at the equatorial region (Figure 8C,D). For ‘Hayward’ kiwifruit, it cannot be confirmed whether the segregation of predicted µ_s_’ was due to granular tissue development around the equator or a difference in flesh firmness, which was also measured around the equator. For the stylar end, it’s possible that shape affected LBI results and thus no difference in predicted µ_s_’ was observed. In the diffusion model described by Farrell et al. [22], the influence of sample curvature is not considered. Qin and Lu calculated a sample size correction factor *sinθ* using spatially resolved diffuse reflectance image and found the corrected µ_s_’ would be less compared with the predicted value because *sinθ* < 1 [26]. Therefore, the actual µ_s_’ should be lower than the predicted µ_s_’. Since ‘SunGold™’ kiwifruit have a more uniform shape and quality parameters, the difference in predicted µ_s_’ was assumed to be due to the granular or water soaking tissue as these CI symptoms usually develop from the stylar end region [16].

When looking at the kiwifruit optical properties, different µ_s_’ was observed for both cultivars with moderate and severe CI in comparison to sound kiwifruit (Figure 8C,D). Such findings might be due to enhanced µ_s_’ caused by the granulated tissue [38]. Different µ_a_ was observed between sound and injured kiwifruit for ‘SunGold™’, and between severe CI and sound-moderate CI for ‘Hayward’ kiwifruit (Figure 8A,B). Thus, it may be possible to use predicted optical properties to segregate kiwifruit with CI, but the large standard deviation found in phantoms may cause classification errors in kiwifruit. Similar results have been observed in other works. A lower transmitted signal intensity of ‘SunGold™’ was observed for fruit with CI when using a dual-laser system [29]. Furthermore, a large variation of µ_a_ and µ_s_’ was reported of kiwifruit skin compared with kiwifruit flesh [32]. Kiwifruit flesh tissue had absorption peaks at 970 nm, 1190 nm and 1390 nm in the NIR region, and the correlation between µ_a_ and SSC reached R² = 0.8 [39]. In the visible region, the peak of green fleshed kiwifruit µ_a_ was reported at 675 nm which is the peak absorption of chlorophyll. The µ_s_’ prediction in kiwifruit showed lower accuracy within 650–750 nm due to high µ_a_ [36,40]. In the present study, high µ_a_ was observed at 520 nm for ‘SunGold™’ (Figure 8) due to the absorption peak of carotenoids. For ‘SunGold™’ kiwifruit optical properties of µ_a_ = 0.1 cm^−1^ and µ_s_’ = 25 cm^−1^ were reported [31], because no pigment absorption took place. Unlike µ_a_, which had absorption peaks for different chemical compositions, kiwifruit µ_s_’ was steadily decreasing [36,40]. Hence, the fruit quality estimation may be more reliable using µ_s_’. Higher µ_s_’ and lower µ_a_ at 632.8 nm was observed for kiwifruit flesh (3.8 and 1.17 cm^−1^) compared to kiwifruit seed part (1.2 and 3.08 cm^−1^) due to different microstructure and pigment contents, respectively [41]. Increased µ_s_’ was observed with decreasing FF, which was explained as new scattering boundaries created when the cell wall was degraded [42].

Reasons for this error might be due to the µ_a_ and µ_s_’ range of the liquid phantoms, because many of the kiwifruit were predicted at the highest class after pre-classification. However, this would indicate a larger range of µ_a_ than reported in previous work. Furthermore, the ratio of µ_a_ and µ_s_’ in kiwifruit does not meet the assumption µ_a_ << µ_s_’ [22].

#### 3.2.3. Segregation of Kiwifruit CI Severity

For ‘SunGold™’ kiwifruit, a high classification accuracy of CI was observed for subsampled sound fruit and CI fruit using LBI parameters (Table 4). Higher classification accuracy of CI was obtained using LBI taken at the stylar end region compared to images acquired at the equator, possibly because the kiwifruit stylar end is the likely location for initial CI development [16]. Using whole fruit LBI parameter information acquired from both locations, the classification accuracy of sound and moderate kiwifruit was slightly improved. This finding indicates that the under-sampling error due to CI symptoms not always appearing in the LBI area was reduced by taking an average of images acquired at multiple locations around the fruit. Therefore, taking multiple measurements on the same fruit is recommended for spot-measurement methods such as LBI to capture the damaged tissue. In a sorting line the fruit are usually rolling, and several spots could be analysed.

Poor classification accuracy was observed for ‘Hayward’ kiwifruit compared with ‘SunGold™’ (Table 4). This could be due to ‘Hayward’ kiwifruit being ungraded thus increasing further variability in having a large variance in size and shape. Furthermore, trichomes could potentially affect the acquisition of diffusion profile.

The advantage of decoupling µ_a_ and µ_s_’ was to separate the information related to chemical composition and cell wall structure. In CI severity detection, µ_s_’ was expected to be correlated with the presence of granular tissue and water soaking tissue, which are the main symptoms differentiating moderate CI and sound kiwifruit.

For the two-class segregation application, to segregate severe CI kiwifruit from sound and moderate CI kiwifruit, the use of LBI parameters achieved a true positive and false-negative rate of 100% and 8% for ‘SunGold™’, and 68% and 23% for ‘Hayward’ considering the same subsampling method (data not shown). However, in real-world situations, CI incidence is low (<5%) when appropriate supply chain conditions are applied. ‘SunGold™’ had a 92% and 75% (Table 5) true positive rate using LBI parameter and µ_a_ and µ_s_’ with the whole sample population in this study (sound and moderate CI and severe CI fruit ratio at 32:1 (*n* = 396). In the subsampled population, the data set is small, but confirms the results. For ‘Hayward’, using the original population (ratio of sound and moderate CI and severe CI fruit = 3.65:1; *n* = 400), only a 39% true positive rate was obtained using LBI parameters and 5% using µ_a_ and µ_s_’ (Table 5) to predict severe CI in kiwifruit. The good model performance for ‘SunGold™’ can be explained by the symptoms of skin discolouration and water soaking of severe CI. These changes of kiwifruit influence µ_a_ while µ_s_’ remained unchanged (Figure 6 and Figure 8A,C). Poor model performance was found for ‘Hayward’ (Figure 7 and Figure 8B,D).

## 4. Conclusions

This work demonstrates that LBI techniques provide a potential non-destructive method for the detection of CI in kiwifruit. The severity of CI could be segregated using optical properties of the fruit (i.e., µ_a_ and µ_s_’). Using a pre-classification model built on liquid phantoms to calibrate the reference matrix of optical properties is a feasible approach to decouple µ_a_ and µ_s_’. The calibration ranges captured the optical properties of kiwifruit providing a reduced effective range compared to earlier successful approaches. The current pre-classification model with the limited effective range has high prediction uncertainties, which may be difficult to overcome. However, for classification questions, the obtained results provide a feasible approach. Future research is required to further improve the pre-classification model, bias correction for fruit curvature, and measurements on several spots on the kiwifruit for application in a sorting line.

## Figures and Tables

**Table 1:** Averaged µ_s_’(cm^−1^) predicted at µ_a_* = 0; µ_s_’(cm^−1^) predicted directly from non-destructive LBI without pre-classification (both open range); µ_s_’(cm^−1^) predicted after pre-classification based on LBI parameters.

µ_s_’*	Range	µ_s_’ (µ_a_* = 0)	µ_s_’ (Open Range)	µ_s_’ (with Pre-Classification)
1.86	0.1–2.5	1.41	3.39	1.11
3.10	2.6–3.6	1.56	3.93	2.61
4.34	3.7–4.8	1.73	4.26	3.57
5.59	4.9–6.5	1.90	4.31	4.36

**Table 2:** Classification accuracy (%) for optical properties (µ_a_ and µ_s_’) of liquid phantoms using laser backscattering image (LBI) profile parameters with the flexible discriminant analysis (FDA) pre-classification model, Farrell-predicted µ_s_’ without pre-classification (open-range) and Farrell-predicted µ_s_’ after pre-classification (pre-classification). Values presented are the average classification accuracy for the 4 µ_s_’ and 8 µ_a_ ranges.

	LBI	Open-Range	Pre-Classification
µ_s_’ (cm^−1^)	87.5	39.3	91.5
µ_a_ (cm^−1^)	65.6	75.0	65.6

**Table 3:** Kiwifruit average flesh firmness (FF) and soluble solids content (SSC) at different chilling injury (CI) severity. Sound means kiwifruit are free from CI symptoms; moderate means kiwifruit with CI symptoms of granulation but no water soaking; severe means kiwifruit with both CI symptoms of granulation and water soaking.

CI Assessment		SunGold™			Hayward	
	*n*	FF (kg_f_) ^1^	SSC (%)	*n*	FF (kg_f_)	SSC (%)
Sound	320	0.88a	15.79a	137	1.21a	15.25a
Moderate	64	0.76b	14.22b	179	0.97b	15.01b
Severe	12	0.74b	12.23c	84	0.57c	14.61c

^1^ Different letter for each cultivar and response variable represents a significant difference by Tukey HSD (*p* < 0.05).

**Table 4:** Classification accuracy (%) of kiwifruit CI severity using LBI parameters and optical properties after subsampling to avoid unbalanced data set with the same number of kiwifruit in each CI category.

	LBI Parameter			µ_a_ and µ_s_’		
	Sound	Moderate	Severe	Sound	Moderate	Severe
‘SunGold™’ (*n* = 36)						
Stylar end	82	71	94	100	0	76
Equatorial region	82	53	82	100	0	18
Whole fruit	83	75	92	100	0	75
‘Hayward’ (*n* = 252)						
Stylar end	49	47	61	76	43	1
Equatorial region	46	42	57	95	8	12
Whole fruit	52	36	58	70	21	35

**Table 5:** Classification true positive accuracy (%) of ‘SunGold™’ and ‘Hayward’ kiwifruit CI severity using all fruit LBI data without subsampling. F_1_ score is the prediction performance indication for severe CI kiwifruit prediction of the unbalanced dataset using accurately predicted and in-correctly predicted severe CI values.

	Sound-Moderate	Severe	F_1_ Score
‘SunGold™’	*n* = 384	*n* = 12	
LBI parameters	98	92	0.73
µ_a_ and µ_s_’	99	75	0.72
‘Hayward’	*n* = 316	*n* = 84	
LBI parameters	97	39	0.53
µ_a_ and µ_s_’	100	5	0.09
